# Stem Cell Therapy for Thyroid Diseases: Progress and Challenges

**DOI:** 10.1016/j.curtheres.2022.100665

**Published:** 2022-03-05

**Authors:** Sunyi Ye, Zhu Lixian Zhu

**Affiliations:** Department of Surgery, The First Affiliated Hospital, School of Medicine, Zhejiang University, Hangzhou, China

**Keywords:** Autoimmune thyroid disorder, Stem cells, Thyroid diseases, Thyroid dysfunction

## Abstract

**Background:**

Thyroid hormones are indispensable for organ development and maintaining homeostasis. Thyroid diseases, including thyroiditis and thyroid cancer, affect the normal secretion of hormones and result in thyroid dysfunction.

**Objective:**

This review focuses on therapeutic applications of stem cells for thyroid diseases.

**Methods:**

A literature search of Medline and PubMed was conducted (January 2000–July 2021) to identify recent reports on stem cell therapy for thyroid diseases.

**Results:**

Stem cells are partially developed cell types. They have the capacity to form specialized cells. Besides embryonic stem cells and mesenchymal stem cells, organ resident stem cells and cancer stem cells are recently reported to have important roles in forming organ specific cells and cancers. Stem cells, especially mesenchymal stem cells, have anti-inflammatory and anticancer functions as well.

**Conclusions:**

This review outlines the therapeutic potency of embryonic stem cells, mesenchymal stem cells, thyroid resident stem cells, and thyroid cancer stem cells in thyroid cells’ regeneration, thyroid function modulation, thyroiditis suppression, and antithyroid cancers. Stem cells represent a promising form of treatment for thyroid disorders.

## Introduction

The thyroid is a critical gland in the endocrine system that secretes hormones. It regulates the metabolism for various kinds of cells and the development of a fetus. Thyroid hormone deficiency causes impaired function of central nervous system, low intelligence quotient, and mental retardation.^1^ Hypothyroidism is caused by diseases that reduce the production of thyroxine. Autoimmune thyroid diseases (AITDs), including fibrotic thyroiditis, are major disorders that cause hypothyroidism. Thyroid malignancies include follicular-derived thyroid cancers (ie, papillary thyroid cancer, follicular thyroid cancer, and anaplastic thyroid cancer) and parafollicular-derived thyroid cancer.^2^ Thyroid autoimmunity is a risk factor of independent of thyroid cancer.^3^ Patients with advanced-stage carcinoma, especially with anaplastic thyroid cancer, lose their surgical opportunities when diagnosis is confirmed. Alternative treatments are urgently needed.

According to the origins, stem cells are divided as embryonic stem cells (ESCs), mesenchymal stem cells (MSCs), organ resident stem cells (RSCs), cancer stem cells (CSCs), perinatal stem cells, and induced pluripotent stem cells (iPSs). MSCs contain stem cells from the connective tissue or stroma surrounding organs and other tissues. Brain, liver, thyroid, and other organs have stem cells themselves, although they account for a relatively small proportion within these organs. The iPSs are developed from mature cells and become ESCs-like cells.^4^ According to the differentiation potency, stem cells may be categorized as totipotent, pluripotent, multipotent, oligopotent, or unipotent cells. The application promising of stem cells is discussed in many fields and often reported in regenerative medicine. Stem cells are capable of developing into organs or tissues, such as livers, hearts, skeletal muscles, and kidneys.^5^ Stem cells are even able to aid in the development of the nervous system^6^; however, the natural development of the organs is quite complex. Thus, the microenvironments required for organ differentiation are difficult to imitate in vitro. The organs or tissues generated from stem cells cannot function as natural ones, because they lack the necessary affiliated cells This is part of the reason why transplant-eligible organs are still in a great demand.^7^ This review summarizes the role of stem cells in thyroid cell directional differentiation, thyroiditis, and thyroid cancers.

## Development of the Thyroid

**Figure 1:**
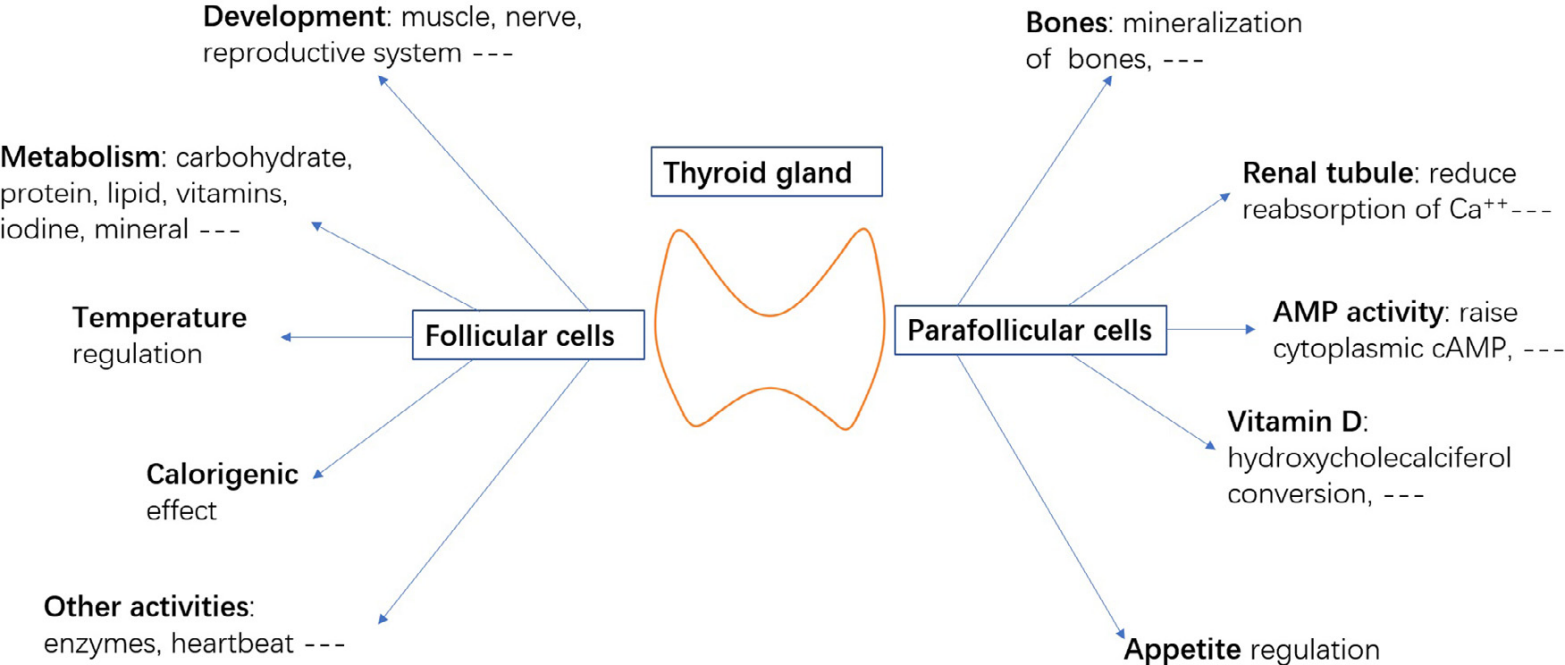
Functions of thyroid follicular cells and parafollicular cells. Ca = calcium ion; cAMP = cyclic adenosine monophosphate.

The development of the fetal thyroid is dependent upon maternal thyroid hormones until the fetal thyroid can secrete enough hormones to support further development. The thyroid contains 2 major functional cell types: follicular cells and parafollicular cells (also called C cells). Fibroblasts, macrophages, and mast cells also inhabit the gland. They are believed to play a role in thyroid pathology. Mast cells can be activated and release specific mediators according to the type of activation.^8^ Follicular cells are derived from endodermal linage. In vitro studies show that Fgf2 (fibroblast growth factor) and Bmp4 are necessary and sufficient to induce murine and human ESCs or iPS directional differentiation.^9^ Other transcription factors, such as Hhex (hematopoietically expressed homeobox), Nkx2-1 (NK2 homeobox 1), Pax8 (Paired box 8), and Foxe1 (Forkhead box E1), are also significant for thyroid development. The network among these 4 factors is elusive. They have mutual regulation on each other, excluding Foxe1.^10^ It is believed that any gene defect inevitably leads to athyreosis or severe hypoplasia.^11^ These gene mutations that cause congenital hypothyroidism account for only a small number of inherited cases, which indicates that other reasons result in congenital hypothyroidism. If left untreated, congenital hypothyroidism causes cretinism. Thyroid hormones have irreplaceable functions in terms of growth, metabolism, and other systems required to maintain homeostasis (see Figure 1).

Parafollicular cells are another crucial type of cell within the thyroid gland. Calcitonin is secreted by parafollicular cells. Calcitonin functions to keep Ca^++^ (Calcium^++^) balance within the body (see Figure 1). For decades, thyroid C cells were hypothesized to be derived from the neural crest^12^; however, lineage tracing was used in mice to pinpoint the origin of C cells in 2015. Researchers found that C cells differentiated from Sox17^+^ (sex determining regionY-box 17) anterior endoderm.^13^ This new finding changes the treatment strategy for C–cell-derived diseases like thyroid medullary cancer. Stem cells can differentiate to follicular cells. Although the differentiation of stem cells to C cells has not been reported, Kwaku et al^14^ recently developed functional thyroid C–cell-like cells from human pluripotent cells. C cells do not attract too much attention compared with follicular cells, but they also have unique characteristics. These 2 major thyroid cells need to be developed and arranged in a physiological manner on a 3-dimensional structure^15^ to work together as an integrated endocrine organ.

## ESCs: An Unlimited Source of Thyroid Cells

The thyroid gland develops from the foregut endoderm. Briefly, under the stimulation of Activin, Noggin, Fgf2, and Bmp4, ESCs differentiate to Nkx2-1 and Pax8 co-expressed endoderm lineage, called progenitor cells. These cells develop to follicles under thyroid stimulating hormone (TSH) stimulation and within a 3-dimensional structure.^11^

Thyroxine is necessary for the development of thyroid gland. Since the early 1990s, researchers have found that thyroid hormone receptor-α and triiodothyronine modulate the neural differentiation.^16^ The specific cell differentiation protocol has not been established. TSH activates the transcription of genes that are necessary for thyroid hormone synthesis and secretion. It also facilitates the transformation of embryonic bodies into thyroid follicular cells.^17^ Although TSH is needed for directional differentiation, other factors also play critical roles throughout the process.

## How Can Endoderm Be Directionally Developed from ESCs?

During the early 2010s, Antonica et al^18^ overexpress transcription factors NKX2-1 and PAX8 in ESCs, which directly form thyroid follicular cells. These induced follicular cells are then grafted into a vascularized niche of a kidney, maintaining thyroid hormone levels in plasma.^18^ In addition to mouse models, functional thyroid follicles may also be induced from the human ESC line.^19^ Further, functional thyroid tissue is developed both in vitro and in vivo using mouse ESCs^20^; however, whether or not human ESCs have developed functional thyroid has not been reported. On the 1 hand, it is not difficult to induce ESCs to differentiate into a single kind of mature cell. On the other hand, it is not easy to establish an entire organ that includes various kinds of functional cells. There are many obstacles that need to be overcome before approaching clinical treatment.^21^ The relationship between ESCs and thyroid carcinoma or thyroiditis has not yet been reported.

## ESC-Conditioned Culture Media Provides Soil to Keep Normal Status

ESC culture supernatant, containing exosomes, has great effects on cell proliferation and differentiation. Mohsin et al^22^ show that mouse ESC-derived exosomes can have therapeutic functions in the heart. They promote cardiomyocyte viability, enhance neovascularization, and reduce cardiac fibrosis in infarcted hearts. These exosomes enrich miR-294 (microRNA-294), which induces cardiac progenitor cell survival and proliferation. Zahra et al^23^ report that ESC-derived exosomes inhibit cardiomyocyte apoptosis induced by chemotherapeutics. They also have anti-inflammatory effects because proinflammatory cytokines are downregulated.^23^ Anti-inflammatory M2 (Macrophage type 2) macrophages can increase after exosome treatment.^24^ From this perspective, exosomes have antifibrotic effects as well. In our primary experiments, we find that ESC-conditioned culture media inhibit liver fibrosis both in vitro and vivo. Sharmin et al^25^ injected ESC-derived macrophages into mice, finding that these macrophages attenuate liver injury and fibrosis. Macrophages secrete similar cytokines contained in exosomes; however, the relationships between ESC-conditioned culture media and thyroid diseases have yet to be reported.

## iPS: From Nonthyroid to Thyroid

IPSs can be derived from mature cells, or even cancer cells, by forcibly expressing stem cell markers such as Oct4 (octamer-binding transcription factor 4), Sox2 (sex determining region Y-box 2), Myc (Myelocytomatosis proteins), and Klf4 (Krüppel like factor 4). These iPSs mimic the properties of ESCs, which means they can differentiate into a wide variety of mature cells. Like ESCs, iPS have great therapeutic promise in organ regeneration, transplantation, disease modeling, anticancer therapy, and genetic modification because little immune rejection occurs. iPSs have been used in cardiac, liver, pancreas, and intestine regeneration, as well as modeling for related diseases.

What about the application of iPSs in thyroid diseases? Ma et al^26^ generated iPS from murine fibroblasts and human dermal fibroblasts. After transfection of Pax8 and Nkx2-1, these iPSs develop to become thyroid cells under stimulation of Activin A and TSH. Thyroid organoids are formed in vitro and then grafted into nude mice. Thyroglobulin expression is confirmed by immunohistology. Under hypoxia conditions, murine iPS cells can differentiate into thyroid cells, expressing thyroid transcription factors and specific markers at different stages. A research team from Japan successfully developed functional thyroid follicular cells from human iPSs. These induced cells secrete free thyroxines in vitro.^27^ This is a great step in the clinical application of iPSs; however, there is still lots of work to do on iPS before clinical treatment. The following questions should be concerned: What is the efficiency of production of iPS? How can the differentiation of iPS be induced directionally? Can mature and functional organs be mimicked using iPS in vitro? What will be the result after the transplantation of cells or organs developed from genetically changed iPS? and, Does genetically changed iPS cause new hereditary diseases?

## MSCs: Immune Regulation and Target Therapy

Compared with ESCs, MSCs have weaker capabilities in terms of the differentiation of special cells. There are few publications about MSCs and thyroid reconstruction. The thyroid gland contains 2 kinds of cells: follicular and parafollicular cells (also called C cells). Most follicular cells are formed during gland development. But what is the origin of the proliferated follicular cells after a partial thyroidectomy? Okamoto et al^13^ traced the origin of newly proliferated follicular cells using the long label-retaining analysis. They found that these cells come from stem cell antigen 1 positive mesenchymal cells.^13^ The epithelial-to-mesenchymal transition plays a critical role in the C–cells-derived cancer, medullary thyroid carcinoma.^28^ Bone marrow-derived MSCs isolated from rats reduce ROS (reactive oxygen species) generation and maintain thyroid function in diabetes mellitus models.^29^ Intravenous injections of bone marrow-derived MSCs isolated ameliorates hypothyroidism-induced pancreatic pathological structure and disturbances.^30^ There is not much data on MSCs and hypothyroidism therapy.

Although the regeneration capability is not as powerful as ESCs, MSCs have their own special properties. First, they can be easily harvested from many kinds of stroma. Bone marrow, adipose tissue, umbilical cord, skeletal muscle, even dental pulp, endometrial polyp, and menstrual blood contain MSCs.^31^ Second, MHC (major histocompatibility complex) antigens on the surface of MSCs are not expressed as often, making them more compatible with immune system. Third, MSCs target injured organ or tissue and show their pro- or anti-inflammatory effect according to the homing niche.^32^ MSCs are believed to be promising for the treatment of immune diseases because of these properties, especially in autoimmune diseases.

AITDs attack functioning follicular cells. Many cytokines and immune cells are involved in AITDs.^33^ This commonly leads to hypothyroidism. Hashimoto's thyroiditis is recognized as an AITD. It is characterized by lymph cell infiltration, fibrotic changes, and parenchymal atrophy.^34^ MSCs, which are isolated from human fetal umbilical cord tissue, are found to have therapeutic functions for Hashimoto's disease in rat models. Fewer thyroid lesions and lymphoid infiltration are observed.^35^ The main mechanism is the modulation of Th17/Treg (T helper cell 17/regulatory T cells) cell balance.^36^ Choi et al^37^ used human adipose tissue derived from MSCs (hATMSC) and murine CLTA4Ig (cytotoxic T lymphocyte-associated antigen-4 immunoglobulin) gene-transduced hATMSC to treat mice with autoimmune thyroiditis. Both hATMSC and gene-transduced hATMSC decrease the production of proinflammatory cytokines and turn the Th1/Th2 (T helper cell 1/ T helper cell 2) balance back again. ATMSC from different kinds of mice have the same treatment effect whether they are syngeneic or allogeneic transplantations.^37^ The intercellular adhesion molecule-1 facilitates the homing process of MSCs after they are injected via veins and modulates the immune cytokines secreted by homed MSCs.^38^ In a rat's Hashimoto's thyroiditis model, MSCs reduce thyroid lesions through lymphoid infiltration via regulating Th17/Treg cells interactions.^36^ Another AITD is Graves’ disease, which usually causes Graves’ ophthalmopathy. Human placental MSCs inhibit the activation of orbital fibroblasts and ameliorate adipogenesis in orbital tissue.^39^^,^^40^

As mentioned above, MSCs migrate to inflammatory sites, but can they house cancer sites? The cancer sites are pathologically similar to those unresolved wounds that induce the migration of MSCs around cancers.^41^ Under magnetic tracing techniques, researchers find that MSCs house various kinds of tumors, acting as both primary and secondary cancer sites for breast cancer,^42^ lung cancer,^43^ ovarian tumors,^44^ and other kinds of malignancies.^45^ As far as we know, the mechanisms for MSCs’ homing to tumor sits are unclear. In breast cancer, it is believed that TGF-β1 (transforming growth factor-β) attracts MSCs into a cancer niche. Neutralizing TGF-β1 antibodies significantly inhibits the migration of MSCs.^42^ In addition to housing injured sites, the chemotactic gradients of cytokines and growth factors, such as macrophage-derived chemokine, stromal-derived factor-1, and insulin-like growth factor-1, play significant roles during the migration.^46^ Jak2/STAT3 (Janus kinases 2/signal transducer and activator of transcription 3), MEK/ERK1/2 (mitogen-activated protein kinase/ extracellular signal-regulated kinase), p38 (mitogen-activated protein kinase p38) signaling pathways, and CXCR4 (C-X-C chemokine receptor type 4) axis are involved in promoting migration of MSCs toward tumor sites.^47^ MSCs differentiate between pro- and anti-inflammatory cell types according to the homing niche microenvironment. They will become tumor-type cells in the cancer environment. Several reports have showed that MSCs promote tumor growth and metastases.^48^ So, how can MSCs be used in anticancer treatments? Because of the self-tumor-homing property, MSCs are used as vectors for specific genes in anticancer therapy. Suicide genes, such as cytosine deaminase, herpes simplex virus thymidine kinase (HSV-TK), CYP2B6/cyclophosphamide, and inducible caspase-9/chemical inducer of dimerization, are transferred into MSCs. Those cells are then injected intravenously.^49^ MSCs with suicide genes inhibit tumor growth^50^ and suppress cancer metastases.^51^ Tang and colleagues^52^ isolated exosomes from MSCs, which inhibit proliferation, invasion, and migration of thyroid carcinoma cells.

## MSC-Conditioned Culture Media Facilitates Regeneration

Exosomes derived from MSCs have anti-inflammatory and antifibrotic functions. Yu et al^53^ report that exosomes derived from GATA-4 overexpressed MSCs preserve cardiac contractile function and reduce infarct size of the heart. The protective effects are mainly exerted by miR-19a (microRNA-19a) in exosomes.^53^ Wang et al^54^ collected exosomes from ESC-induced MSCs (ESC-MSCs) and found that the intra-articular injection of these exosomes alleviate cartilage destruction and degradation, which attenuates osteoarthritis.^54^ Human ESC–MSCs-derived molecules exhibit therapeutic properties in acute liver injury models.^55^ MSC-conditioned media increases the number of Th2 and Treg cells while reducing the number of Th17 cells, which ameliorates fulminant hepatic failure and reduces chronic liver fibrosis in vivo.^55^ Sahoo et al^56^ harvested exosomes from human CD34+ (Cluster of differentiation 34 positive) conditioned culture media, finding that these exosomes promote endothelial cell proliferation and viability in vitro. They also have the capacity to stimulate angiogenesis in vivo.^56^ Exosomes are promising in the cell-free therapeutic strategy for future studies. Recently, Wen et al^57^^,^^58^ reported that mouse ESC complementation induces thyroid morphogenesis in Nkx2-1^−/−^ embryos (Nkx2-1 gene knockout embryos mice) and Fgf10 Ex1mut/Ex3mut mice (Fgf10 Ex1 genes mutation mice/Ex3 gene mutation mice). These generated tissues have normal morphology and physiological function.^57^^,^^58^

## Thyroid RSCs: Local Pool for Functional Cells

Self-regenerative organs or tissues are believed to have RSCs and precursor cells, especially within the liver, skin, and inner surface of the intestine. The thyroid has a low rate of turnover, with only 4 to 8 renewals over the course of a lifetime. In partial thyroidectomy models, it has also been found that thyroid glands have the capability of hypertrophy and hyperplasia.^59^ Thyroid resident precursor cells inhabit the gland; however, these cells account for a small proportion in a physiological status. It is estimated that 1 out of 1000 cells are stem or precursor cells.^60^ Hoshi et al^61^ isolated side population (SP) cells from mice thyroids. SP cells are divided into 2 subgroups according to the expression of stem cell antigen 1 (Sca1). CD45^–^/c-KIT^–^/Sca1^+^ (Cluster of differentiation 45 gene negative/Receptor tyrosine-protein kinase) is SP1, whereas CD45^–^/c-KIT^–^/Sca1^–^ is SP2. They both highly express the stem cell markers, Oct4 and ABCG2 (ATP-binding cassette G2). In human thyroid glands, Oct4^+^ cells are also found, expressing Abcg2 (ATP-binding cassette G2), Gata4 (GATA gene-binding protein 4), and Hfn4α (hepatocytes nuclear factor 4-α).^62^ The latter 2 are endodermal lineage markers. Although SP cells have both stem and endodermal markers, they do not express thyroglobulin and TPO (Thyroid Peroxidase) genes. Under certain stimuli, they can differentiate into normal thyrocytes. The question is whether these precursor cells reside within the gland^61^ or migrate from other organs, such as bone marrow, upon stimulation. Both resident and migrated stem cells are involved, keeping the thyroid in a physiological status. Recently, thyroid RSCs are recognized by expressing 2 major transcription factors, Pax-8 and NKX2-1(TTF1).^63^ Both play critical roles in thyroid regeneration after severe damage.^64^

**Figure 2:**
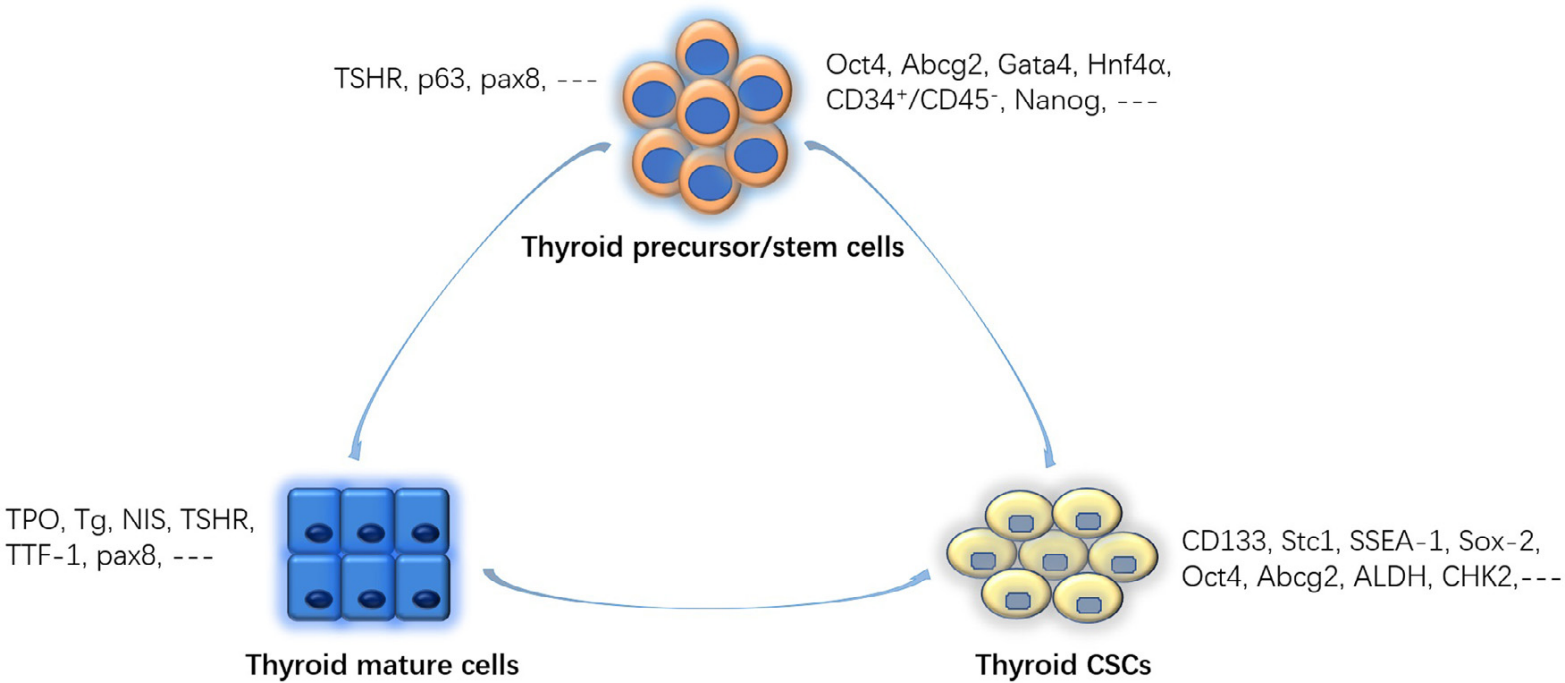
Molecular markers express on thyroid mature cells, thyroid precursor/stem cells, and thyroid cancer stem cells. They share some markers. CSC = cancer stem cells.

Stem cells within the thyroid may be promising in cell repair and anticancer therapy (see Figure 2). Under certain circumstances, RSCs will transform to functional thyroid cells, replacing those destroyed cells caused by injury or inflammation.^65^ Local transplantation of stem cells into target organ or tissue is routinely used, whereas conditioned culture media or exosomes from various stem cells may be an alternative option for repair and anticancer treatment.

## Thyroid CSCs: An Origin of Thyroid Cancer Cells?

There are 3 models for carcinogenesis of the thyroid gland: multiple mutations accumulation model, fetal cell carcinogenesis model, and the CSC model. The last model is now widely accepted. CSCs are believed to have critical functions in carcinoma initiation, metastasis, recurrence, and drug resistance. Where are these CSCs from? The cellular origin of CSCs from the thyroid is unclear. There are several hypotheses regarding origination. First, they may come from resident normal stem cells. Under certain stimulation, part of RSCs may transform into CSCs. Second, some functional thyroid cells may become CSCs, mimicking the process of iPS. Ma et al^66^ report CSCs come from thyroid cells via epithelial–mesenchymal transition.^66^ Third, they are believed to be derived from some other precursor cells. A dynamic theory has been proposed. It is said that there is a balance between CSC and non-CSC populations. They interconvert to each other under certain conditions. It is difficult to distinguish between normal RSCs and CSCs. Biomarker distributions may have some differences. Sca1, Oct4, homeobox transcription factor Nanog, paired box gene 8, thyroid transcription factor 1, and TIF-2 are commonly expressed in normal RSCs. CD133 (prominin-1), Oct4, stanniocalcin 1, once-fetal fibronectin, aldehyde dehydrogenase,^67^ and cell cycle checkpoint kinase 2 usually present in CSCs. However, it needs to be acknowledged that many markers are both expressed in RSCs and CSCs. Oct4 is a stemness marker, whereas Abcg2, multidrug resistance-associated protein 1, and multidrug resistance 1 are drug-resistance markers. That is why CSCs are not sensitive to chemo drugs. Dai et al^68^ reported that long noncoding RNA, DOCK9-AS2, which is isolated from thyroid CSC-like cells, promotes migration and invasion of papillary thyroid carcinoma.

## Gene Therapy Strategy: The Future of Thyroid Cancers

Genes are inserted into vectors targeting tumor-specific cells. Gene therapy strategies typically refer to 3 different types: carrying enzyme/prodrug systems, toxic genes, and proapoptotic genes. The commonly used enzyme/prodrug combination is herpes simplex virus thymidine kinase (HSV-TK) and ganciclovir. Multiple chemical reactions are involved in the anticancer system. Briefly, HSV-TK converts deoxythymidine into deoxythymidine monophosphate, which then becomes deoxythymidine triphosphate under stimulation of cellular kinase. Ganciclovir converts to ganciclovir monophosphate under thymidine kinase. Ganciclovir monophosphate is further dephosphorylated into ganciclovir triphosphate, which causes termination of DNA chain replication. Cell apoptosis occurs after the treatment of HSV-TK and ganciclovir. Other therapeutic combinations include cytosine deaminase and 5-fluorocytosine, purine nucleoside phosphorylase and 6-methylpurine deoxy riboside or fludarabine, horseradish peroxidase, and indole-3-acetic acid.^69^ Second, the toxic genes, such as diphtheria toxin, streptolysin O, anthrax toxin, cholera toxin B, apoptin, saporin, and alpha-holin gene, are used to kill off various cancers. Vectors carrying these toxic genes target tumor cells and cause apoptosis or death.^70^ Third, proapoptotic genes induce cell suicide. P53, caspase 3, caspase 9, Bcl-2 gene family,^50^ and TRAIL^51^ are inserted into vectors and then carried into target tumors.

Vectors carrying those genes are developed from various sources. Viral vectors, synthetic vectors, and cell-derived vectors are the 3 major carriers. Retroviruses, lentiviruses, and adenoviruses are commonly applied as vectors. Viral vectors are highly efficient; however, safety is a concern. Synthetic vectors have little toxicity and immunogenicity. The directional movement of these vectors is the problem. Stem cells are promising as gene vectors. In particular, after the development of iPS, immunogenicity is no longer a big problem. Recently, cotransfection with 2 suicide genes was used to increase the successful rate of gene therapy.

Anaplastic thyroid cancer (ATC) is the most malignant tumor. Patients with ATC usually lose their opportunity for surgery when they are diagnosed. It is crucial to find an alternative way to treat ATC. Senthilkumar et al^71^ used a synthetic tetracycline-on switch system to control the expression of thymidine kinase. Then, a retroviral vector expressed HSV thymidine kinase (ie, HSV1-sr39TK) was developed and transduced into MSCs. Those MSCs are co-cultured with the ATC cell line, which has decreased viability; however, the therapeutic effect of transduced MSCs in patients with ATC need to be confirmed in vivo.

## Conclusions

**Figure 3:**
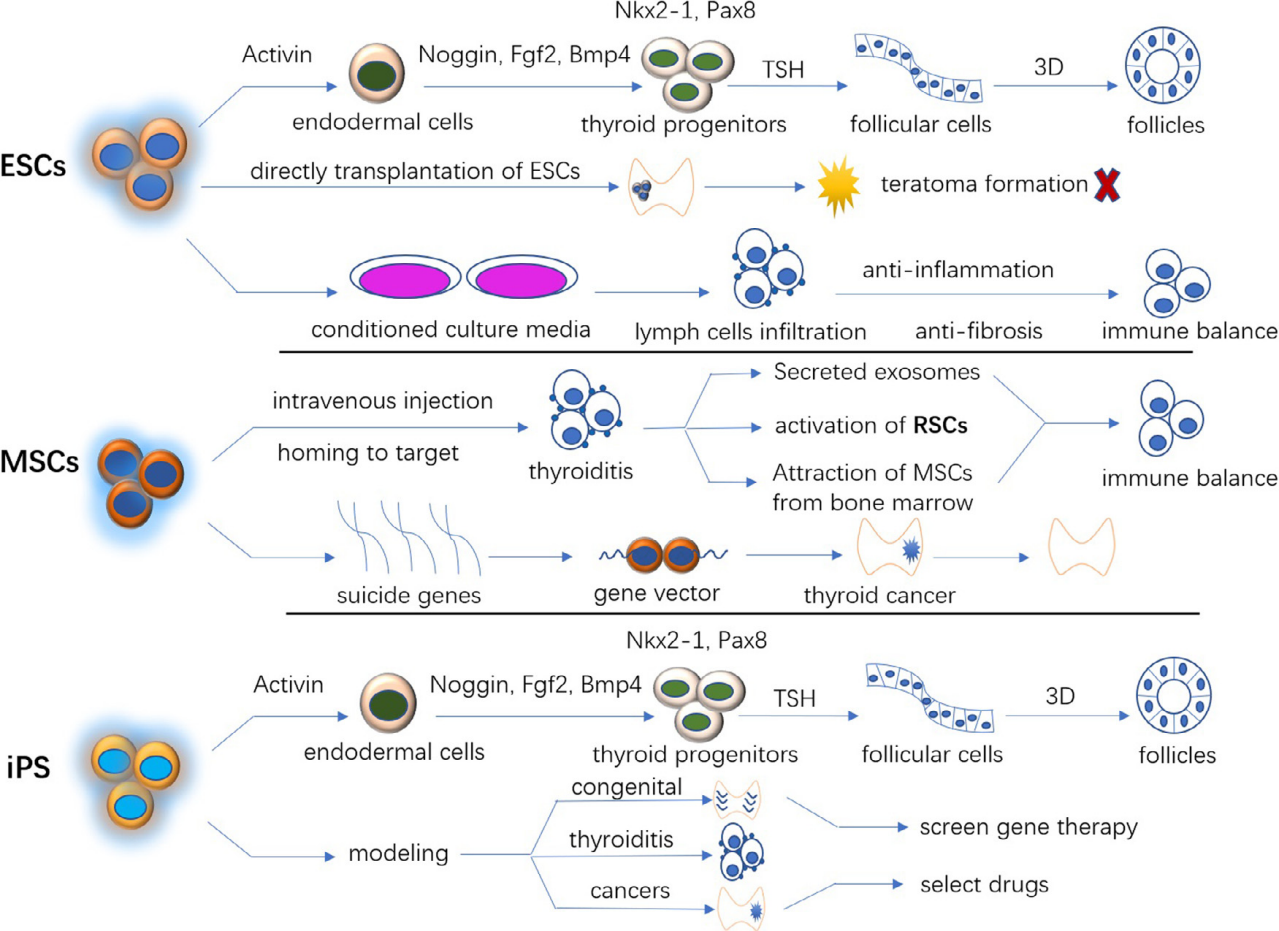
The roles of stem cells in the treatment of thyroid diseases. 3D = xxxxx (three dimensions); ESC = embryonic stem cells; iPS = induced pluripotent stem cells; MSC = mesenchymal stem cells; RSC = resident stem cells; TSH = thyroid stimulating hormone.

Stem cells, characterized by multilineage differentiation and proliferation, are thought to have the capability to make up these deficiencies (see Figure 3). ESCs have teratoma formation property,^72^ conditioned culture media is an alternative use of stem cells. Induced pluripotent stem cells are recently man-made cells mimicking the characteristics of ESCs. MSCs are used the most in life science field compared to other stem cells. They inhibit lymphoid infiltration in autoimmune thyroiditis and attenuate the adipogenesis in orbital tissue caused by Graves’ disease. Stem cells resident in the thyroid organ are precursors of mature thyroid cells. They are thought to renew senescent cells and maintain thyroid in a physiological status. Cancer stem cells are commonly thought to be the initiation of carcinogenesis. Precisely recognizing and disabling the activation of these CSCs will be essential for reversing carcinogenesis. he multipotent properties of stem cells bring multiple possibilities. Stem cell treatment in thyroid diseases requires further examination.
